# Association between symptoms of severe periodontitis and post-bronchodilator lung function: results from the China pulmonary health study

**DOI:** 10.1186/s12890-023-02485-6

**Published:** 2023-06-17

**Authors:** Zhiqiang Liu, Xuan Zhou, Lirong Liang, Xiaozhe Han, Ting Yang, Kewu Huang, Yingxiang Lin, Zuomin Wang, Chen Wang

**Affiliations:** 1 Department of Stomatology, Beijing Chao-Yang Hospital, Capital Medical University, 8 Gongti South Road, Chaoyang District, Beijing, 100020 China; 2 Department of Clinical Epidemiology, Beijing Chao-Yang Hospital, Capital Medical University, Beijing, China; 3 Beijing Institute of Respiratory Medicine, Beijing, China; 4 Department of Oral Science and Translational Research, Nova Southeastern University College of Dental Medicine, Florida, USA; 5 Department of Pulmonary and Critical Care Medicine, Center of Respiratory Medicine, China-Japan Friendship Hospital, 2 Yinghuayuan Dongjie, Chaoyang District, Beijing, 100029 China; 6 National Clinical Research Center for Respiratory Diseases, Beijing, China; 7 Institute of Respiratory Medicine, Peking Union Medical College, Beijing, China; 8 Department of Respiratory Medicine, Capital Medical University, Beijing, China; 9 Department of Pulmonary and Critical Care Medicine, Beijing Key Laboratory of Respiratory and Pulmonary Circulation Disorders, Beijing Chao-Yang Hospital, Beijing, China; 10 WHO Collaborating Center for Tobacco Cessation and Respiratory Diseases Prevention, Beijing, China

**Keywords:** Lung function, Periodontitis, COPD, Cross-sectional, Epidemiology, Risk factor

## Abstract

**Background:**

The association between periodontitis and post-bronchodilator lung function is unclear. We aimed to determine the associations between symptoms of severe periodontitis (SSP) and post-bronchodilator lung function in the Chinese population.

**Methods:**

A cross-sectional study (China Pulmonary Health study) was conducted from 2012 to 2015 in a large Chinese nationally representative sample of 49,202 participants aged 20–89 years. Data on demographic characteristics and periodontal symptoms of participants were collected by questionnaire. Participants who had at least one of the two severe symptoms (tooth mobility and natural tooth loss) in the past year were defined to have SSP, which was set as one variable for analyses. Post-bronchodilator lung function data including forced expiratory volume in 1 s (FEV_1_) and forced vital capacity (FVC) were collected by spirometry.

**Results:**

The values of post-FEV_1_, post-FVC and post-FEV_1_/FVC of the participants with SSP were all significantly lower than the participants without SSP (all *p* < 0.001). SSP were significantly associated with post-FEV_1_/FVC < 0.7 (*p* < 0.001). In the multiple regression analyses, SSP were still negatively associated with post-FEV_1_(b = -0.04, 95%CI (-0.05 -0.03), *p* < 0.001), post-FEV_1_/FVC (b = -0.45, 95%CI (-0.63, -0.28), *p* < 0.001) and significantly associated with post-FEV_1_/FVC < 0.7 (OR = 1.08, 95%CI 1.01—1.16, *p* = 0.03) after full adjustment for potential confounders.

**Conclusions:**

Our data suggest that SSP were negatively associated with post-bronchodilator lung function in the Chinese population. Longitudinal cohort studies are needed to confirm these associations in the future.

## Background

Periodontitis is a chronic infection and inflammatory disease that is initially caused by periodontal pathogens infection and eventually leads to alveolar bone resorption, tooth mobility and tooth loss. The worldwide prevalence of severe periodontitis is 10.8% [1]. According to the 4th Chinese national oral health epidemiological survey, almost 90% of Chinese adults suffered from periodontal disease of various severities, and about 30% had severe periodontitis (Stage III and IV) in the population aged 35–74 years [2]. Although the infection and inflammation mainly manifest in the local periodontal tissue, periodontitis has been found to be associated with many systemic diseases such as cardiovascular disease, diabetes mellitus, rheumatoid arthritis, chronic kidney disease and Alzheimer's disease [3].

In the past decades, increased evidence demonstrated that periodontitis was associated with obstructive lung diseases including chronic obstructive pulmonary disease (COPD) [4]. Obstructive impaired lung function is the key feature of COPD, which can be determined by spirometry [5]. Several cross-sectional studies with relatively small sample sizes have assessed the potential association between periodontal health and impaired lung function previously [6–9]. Although these studies used different periodontal and lung function parameters to evaluate periodontal health and lung function, the results all showed that poor periodontal health was associated with airflow limitation or reduced lung function [6–9]. Recently, two large cross-sectional studies based on the national population of Korea and the United States have assessed the association between periodontitis and impaired lung function [10, 11]. However, the results showed that periodontitis was not significantly associated with obstructive lung function impairment which was defined as forced expiratory volume in 1 s (FEV_1_)/forced vital capacity (FVC) < 0.7 [10, 11]. This inconsistency may be partly attributed to the different study populations. Therefore, the association between periodontitis and impaired lung function needs to be further clarified in large population-based studies.

The bronchodilator test is used to help diagnose obstructive airway diseases such as COPD and asthma by measuring the reversibility of obstructive impaired lung function after inhalation of bronchodilators [12]. The Global Initiative for Chronic Obstructive Lung Disease (GOLD) guidelines defined COPD as FEV_1_/FVC < 0.7 from a post-bronchilator test [13]. A previous study used data on pre-bronchodilator lung function to access the association between periodontitis and lung function [8], and the others did not describe the lung function data were collected pre- or post-bronchodilator [6, 7, 9–11]. Therefore, whether periodontitis is associated with post-bronchodilator lung function needs to be studied.

Thus, we designed this study by using data from a nationally representative sample (57,779 subjects) of Chinese adults from a large nation-wide cross-sectional survey (China Pulmonary Health Study, CPHS) [14]. In this study, we aimed to determine the associations between symptoms of severe periodontitis (SSP) and post-bronchodilator lung function in the Chinese population.

## Materials and methods

### Study population

**Fig. 1:**
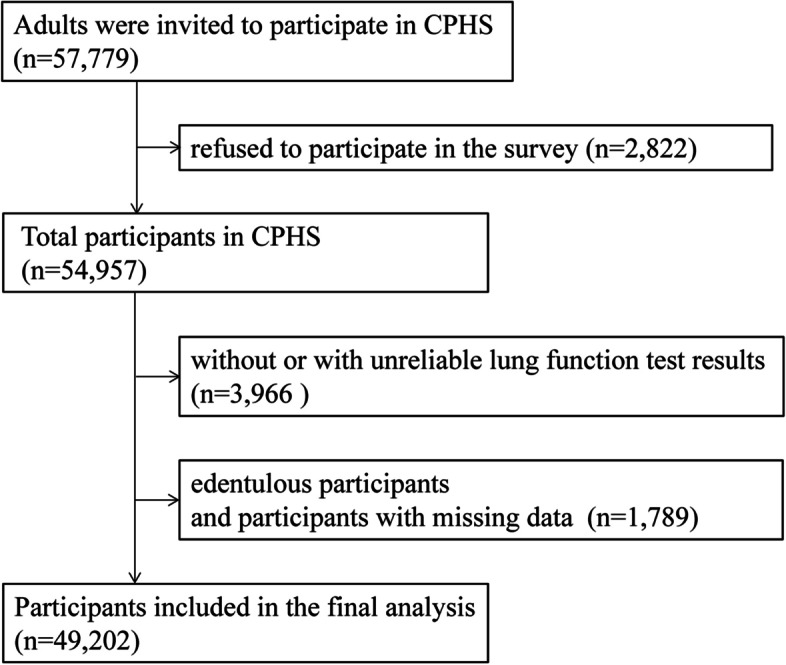
The flow diagram of the selection of study participants

The CPHS was a cross-sectional study in a nationally representative sample of adults aged 20-89y conducted in China from 2012 to 2015 [14]. The study was designed to investigate the pulmonary health status of the Chinese population. Information related to pulmonary health, such as environmental factors, psychological factors and other related systemic health status including oral health, were also collected in this study. The study was approved by the ethics review committee of Beijing Capital Medical University and other participating institutes, and was conducted in accordance with the Helsinki Declaration of 1975, as revised in 2013. Written informed consent was obtained from all study participants. Details of the study design have been published previously [14]. Briefly, 57,779 individuals which were enrolled by a multistage stratified cluster sampling procedure were invited to participate in nation-wide, of whom 49,202 had reliable lung function test results, with natural teeth in the mouth and with no missing data were included in this study. The flow diagram of the selection of study participants is shown in Fig. 1.

### Symptoms of severe periodontitis (SSP)

As a part of the CPHS, trained interviewers asked all participants whether they had experienced SSP in the past year, including tooth mobility and natural tooth loss. The specific questions that interviewers asked were “Have you had loose teeth in the past year?”, and “Have you had spontaneous tooth loss in the past year?”. The answers for these two questions were both set as “Yes” or “No”. Participants who answered “Yes” for at least one of the two questions were defined as having SSP, which was set as one variable for analyses in this study.

### Lung function variables

Trained and certified technicians performed pulmonary function tests on all participants with a MasterScreen Pneumo PC spirometer (CareFusion, Yorba Linda, CA, USA). Details of the pulmonary function tests have been published previously [14]. An expert panel was responsible for quality assurance based on the American Thoracic Society and European Respiratory Society criteria [15], and poor quality tests were excluded.

The values of the ventilation function variables FEV_1_ and FVC after bronchodilator inhalation of all participants were collected. Post-FEV_1_/FVC was calculated and post-FEV_1/_FVC ≥ 0.7 and < 0.7 were set as categorical variables according to the GOLD guideline [13].

### Other variables

Trained interviewers administered standardized questionnaires containing information on demographic characteristics, education background, smoking status, residence, biomass fuel exposure, occupational exposure, chronic cough during childhood, parental history of respiratory diseases, history of respiratory diseases and other systemic diseases. We divided the participants into three groups (20-39y, 40-59y and ≥ 60y) according to the age. We categorized the education background as “primary school and lower”; “middle and high school” and “college and higher”; and categorized the smoking status as “never smoker” and “current/former smoker”. Never smokers were defined as those who had smoked fewer than 100 cigarettes in their lifetime and did not currently smoke. Residence was categorized as “urban” and “rural”. Biomass fuel exposure was defined as using woody fuel or animal waste for cooking or heating during the past 6 months or longer. Occupational exposure was defined as people engaged in work with dust exposure such as mining, quarrying, textile and cotton for more than 3 months. History of chronic bronchitis was defined as cough and sputum production for at least 3 months in each of two consecutive years. We categorized chronic cough during childhood before age 14 years as frequent (cumulative > 3 months per year), sometimes (1–3 months per year), and rare (< 1 month per year). Participants and their parents with one or more of the following conditions were considered to have respiratory disease, including COPD, emphysema, chronic bronchitis, bronchiectasis, asthma, tuberculosis, idiopathic pulmonary fibrosis and lung cancer. Participants with one or more of the following conditions were considered to have systemic disease, including stroke, coronary heart disease, hypertension, diabetes mellitus, depression, osteoporosis and anemia. Body height and weight were determined using calibrated scales, and body mass index (BMI) was calculated as weight/ height squared.

### Statistical analyses

Normally distributed continuous variables were expressed as mean ± sd. Categorical variables were presented as n%. For continuous variables, independent t-test or one-way ANOVA was used, and chi-square test was used for categorical variables in univariate analyses. SPSS statistical software (version 20.0; SPSS Inc., Chicago, IL, USA) was used for the data analyses, and statistical significance was considered at a two-sided *p* < 0.05.

Multiple linear regression analyses were performed to calculate the unstandardized coefficients (b) and 95% confidence interval (CI) for the associations between SSP and continuous lung function variables. Multiple logistic regression analyses were performed to calculate the odds ratios (OR) and 95%CI for the associations between SSP and FEV_1/_FVC (≥ 0.7 and < 0.7). The adjusted model 1 was adjusted for age, gender, height, body mass index, education, smoking status, residence, occupational exposure and biomass fuel exposure. The adjusted model 2 was additionally adjusted for chronic cough during childhood, parental history of respiratory diseases, history of chronic bronchitis, other respiratory diseases and other systemic diseases.

In order to exclude effects of the confounding factors for lung function, subgroup analyses were performed for the associations between SSP and post-FEV_1_ and post-FEV_1_/FVC by multiple linear regression. The subgroup analyses were separately stratified by age, gender, smoking status, residence, history of chronic bronchitis and other respiratory diseases, which were grouped as described above. The multiple linear regression was adjusted for age, gender, height, body mass index, education, smoking status, residence, occupational exposure, biomass fuel exposure, chronic cough during childhood, parental history of respiratory diseases, history of chronic bronchitis, other respiratory diseases and other systemic diseases. When the study population was grouped by a variable, this variable was not included in the adjusted model.

## Results

The demographic characteristics of the total 49,202 participants are shown in Table 1 and 2. Age, height, BMI, education, smoking status, residence, biomass fuel exposure, occupational exposure, parental history of respiratory diseases, history of chronic bronchitis, other respiratory diseases and other systemic diseases were all significantly associated with SSP (all *p* < 0.001, Table 1). And all demographic characteristics were all significantly associated with post-FEV_1_/FVC < 0.7 (all *p* < 0.001, Table 2).

**Table 1:** Demographic characteristics of the study population grouped by symptoms of severe periodontitis

**Characteristics**	**Symptoms of severe periodontitis**		**Total** *N* = 49,202	***p* value**
	**Yes** *N* = 10,860 (22.07%)	**No** *N* = 38,342 (77.93%)		
**Age (n, %)**				< 0.001
20–39 y	725 (6.70%)	11,583 (30.20%)	12,308 (25.00%)	
40–59 y	5257 (48.40%)	19,225 (50.10%)	24,482 (49.80%)	
≧ 60 y	4878 (44.90%)	7534 (19.60%)	12,412 (25.20%)	
Mean age (year, mean (sd))	57.45 (11.30)	47.15 (13.62)	49.43 (13.82)	
**Gender (n, %)**				0.27
Male	4614 (42.5%)	16,062 (41.9%)	20,676 (42.0%)	
Female	6246 (57.5%)	22,280 (58.1%)	28,526 (58.0%)	
**Height** (meter, mean (sd))	160.37 (8.32)	161.82 (8.27)	161.50 (8.30)	< 0.001
**BMI (n, %)**				< 0.001
< 18.5 (underweight)	376 (3.5%)	1601 (4.2%)	1977 (4.0%)	
18.5–25.0 (normal weight)	6335 (58.3%)	23,280 (60.7%)	29,615 (60.2%)	
≥ 25.0 (overweight & obese)	4149 (38.2%)	13,461 (35.1%)	17,610 (35.8%)	
Mean BMI (kg/m2) (mean,sd)	24.24 (3.47)	23.92 (3.52)	23.99 (3.51)	
**Education (n, %)**				< 0.001
Primary school and lower	4012 (36.9%)	8379 (21.9%)	12,391 (25.2%)	
Middle and high school	5840 (53.8%)	22,269 (58.1%)	28,109 (57.1%)	
College and higher	1008 (9.3%)	7694 (20.1%)	8702 (17.7%)	
**Smoking status (n, %)**				< 0.001
Never smoker	7424 (68.4%)	27,716 (72.3%)	35,140 (71.4%)	
Current/Former smoker	3436 (31.6%)	10,626 (27.7%)	14,062 (28.6%)	
**Residence (n, %)**				< 0.001
Urban	6745 (62.1%)	24,994 (65.2%)	31,739 (64.5%)	
Rural	4115 (37.9%)	13,348 (34.8%)	17,463 (35.5%)	
**Occupational exposure (n, %)**				< 0.001
Yes	2960 (27.3%)	9061 (23.6%)	12,021 (24.4%)	
No	7900 (72.7%)	29,281 (76.4%)	37,181 (75.6%)	
**Biomass fuel exposure (n, %)**				< 0.001
Yes	3767 (34.7%)	9600 (25.0%)	13,367 (27.2%)	
No	7093 (65.3%)	28,742 (75.0%)	35,835 (72.8%)	
**Chronic cough during childhood (age < 14 years) (n, %)**				0.62
Rare	9756 (89.8%)	34,381 (89.7%)	44,137 (89.7%)	
Sometimes/Frequent	1104 (10.2%)	3961 (10.3%)	5065 (10.3%)	
**Parental history of respiratory diseases (n, %)**				< 0.001
Yes	2103 (19.4%)	6208 (16.2%)	8311 (16.9%)	
No	8757 (80.6%)	32,134 (83.8%)	40,891 (83.1%)	
**History of chronic bronchitis (n, %)**				
Yes	546 (5.0%)	1054 (2.7%)	1600 (3.3%)	< 0.001
No	10,314 (95.0%)	37,288 (97.3%)	47,602 (96.7%)	
**Other respiratory diseases (n, %)**				
Yes	404 (3.7%)	833 (2.2%)	1237 (2.5%)	< 0.001
No	10,456 (96.3%)	37,509 (97.8%)	47,965 (97.5%)	
**Other systemic diseases (n, %)**				< 0.001
Yes	2103 (19.4%)	4010 (10.5%)	6113 (12.4%)	
No	8757 (80.6%)	34,332 (89.5%)	43,089 (87.6%)	

*BMI* Body mass index. Other respiratory diseases included chronic obstructive pulmonary disease, emphysema, bronchiectasis, asthma, tuberculosis, idiopathic pulmonary fibrosis and lung cancer. Other systemic diseases included stroke, coronary heart disease, hypertension, diabetes mellitus, depression, osteoporosis and anemia. *p*-values were calculated by t-test for continuous variables and chi-square test for categorical variables

**Table 2:** Demographic characteristics of the study population grouped by post-FEV_1_/FVC < 0.7 and ≥ 0.7

**Characteristics**	**Post-FEV**_**1**_**/FVC < 0.7** *N* = 4,759 (9.67%)	**Post-FEV**_**1**_**/FVC ≥ 0.7** *N* = 44,443 (90.33%)	***p* value**
**Age (n, %)**			< 0.001
20–39 y	228 (4.8%)	12,080 (27.2%)	
40–59 y	1834 (38.5%)	22,648 (51.0%)	
≧ 60 y	2697 (56.7%)	9715 (21.9%)	
Mean age (year, mean (sd))	60.36 (11.39)	48.25 (13.54)	
**Gender (n, %)**			< 0.001
Male	2969 (62.4%)	17,707 (39.8%)	
Female	1790 (37.6%)	26,736 (60.2%)	
**Height** (meter, mean (sd))	162.17 (8.54)	161.42 (8.28)	
**BMI (n, %)**			< 0.001
< 18.5 (underweight)	231 (4.9%)	1746 (3.9%)	
18.5–25.0 (normal weight)	2927 (61.5%)	26,688 (60.0%)	
≥ 25.0 (overweight & obese)	1601 (33.6%)	16,009 (36.0%)	
Mean BMI (kg/m2) (mean,sd)	23.78 (3.48)	24.01 (3.52)	
**Education (n, %)**			< 0.001
Primary school and lower	2008 (42.2%)	10,383 (23.4%)	
Middle and high school	2341 (49.2%)	25,768 (58.0%)	
College and higher	410 (8.6%)	8292 (18.7%)	
**Smoking status (n, %)**			< 0.001
Never smoker	2406 (50.6%)	32,734 (73.7%)	
Current/Former smoker	2353 (49.4%)	11,709 (26.3%)	
**Residence (n, %)**			< 0.001
Urban	2952 (62.0%)	28,787 (64.8%)	
Rural	1807 (38.0%)	15,656 (35.2%)	
**Occupational exposure (n, %)**			< 0.001
Yes	1302 (27.4%)	10,719 (24.1%)	
No	3457 (72.6%)	33,724 (75.9%)	
**Biomass fuel exposure (n, %)**			< 0.001
Yes	1661 (34.9%)	11,706 (26.3%)	
No	3098 (65.1%)	32,737 (73.7%)	
**Chronic cough during childhood (age < 14 years) (n, %)**			< 0.001
Rare	4125 (86.7%)	40,012 (90.0%)	
Sometimes/Frequent	634 (13.3%)	4431 (10.0%)	
**Parental history of respiratory diseases (n, %)**			< 0.001
Yes	1021 (21.5%)	7290 (16.4%)	
No	3738 (78.5%)	37,153 (83.6%)	
**History of chronic bronchitis (n, %)**			< 0.001
Yes	516 (10.8%)	1084 (2.4%)	
No	4243 (89.2%)	43,359 (97.6%)	
**Other respiratory diseases (n, %)**			< 0.001
Yes	491 (10.3%)	746 (1.7%)	
No	4268 (89.7%)	43,697 (98.3%)	
**Other systemic diseases (n, %)**			< 0.001
Yes	814 (17.1%)	5299 (11.9%)	
No	3945 (82.9%)	39,144 (88.1%)	
**Symptoms of severe periodontitis (n, %)**			< 0.001
Yes	1604 (33.7%)	9256 (20.8%)	
No	3155 (66.3%)	35,187 (79.2%)	

*BMI* Body mass index. Other respiratory diseases included chronic obstructive pulmonary disease, emphysema, bronchiectasis, asthma, tuberculosis, idiopathic pulmonary fibrosis and lung cancer. Other systemic diseases included stroke, coronary heart disease, hypertension, diabetes mellitus, depression, osteoporosis and anemia. *p*-values were calculated by t-test for continuous variables and chi-square test for categorical variables

The values of post-FEV_1_, post-FVC and post-FEV_1_/FVC of the participants with SSP were all significantly lower than the participants without SSP (all *p* < 0.001). SSP were significantly associated with post-FEV_1_/FVC < 0.7 (*p* < 0.001) (Table 3).

**Table 3:** Post-bronchodilator lung function of the study population

**Lung function variables** (mean (sd) or n (%))	**Symptoms of severe periodontitis**		**Total**	***p* value**
	**Ye**	**No**		
Post-FEV_1_ (liter)	2.49 (0.69)	2.82 (0.76)	2.75 (0.75)	< 0.001
Post-FVC (liter)	3.20 (0.84)	3.47 (0.88)	3.41 (0.88)	< 0.001
Post-FEV_1_/FVC (%)	78.32 (9.75)	81.45 (8.71)	80.76 (9.04)	< 0.001
Post-FEV_1_/FVC < 0.7	1604 (14.8%)	3155 (8.2%)	4759 (9.7%)	< 0.001

*Post* After bronchodilator inhalation, *FEV*_*1*_ Forced expiratory volume in 1 s, *FVC* Forced vital capacity. *p*-values were calculated by t-test for continuous variables and chi-square test for categorical variables

In the multiple regression analyses, SSP were still negatively associated with post-FEV_1_(b = -0.05, 95%CI (-0.06, -0.04), *p* < 0.001 and b = -0.04, 95%CI (-0.05 -0.03), *p* < 0.001), post-FEV_1_/FVC (b = -0.56, 95%CI (-0.74, -0.38), *p* < 0.001 and b = -0.45, 95%CI (-0.63, -0.28), *p* < 0.001) and significantly associated with post-FEV_1_/FVC < 0.7 (OR = 1.11, 95%CI 1.05—1.19, *p* = 0.001 and OR = 1.08, 95%CI 1.01—1.16, *p* = 0.03) both in the adjusted model 1 and model 2 (Table 4).

**Table 4:** Association between symptoms of severe periodontitis and post-bronchodilator lung function by multiple regression analyses

Lung function variables	Symptoms of severe periodontitis (yes vs. no)					
	**Unadjusted model**		**Adjusted model 1**		**Adjusted model 2**	
	**b or OR (95%CI)**	***p* value**	**b or OR (95%CI)**	***p* value**	**b or OR (95%CI)**	***p* value**
Post-FEV_1_	-0.33 (-0.34, -0.31)	< 0.001	-0.05 (-0.06, -0.04)	< 0.001	-0.04 (-0.05, -0.03)	< 0.001
Post-FEV_1_/FVC	-3.13 (-3.32, -2.94)	< 0.001	-0.56 (-0.74, -0.38)	< 0.001	-0.45 (-0.63, -0.28)	< 0.001
Post-FEV_1_/FVC < 0.7	1.93 (1.81, 2.06)	< 0.001	1.11 (1.04, 1.19)	0.003	1.08 (1.01, 1.16)	0.03

*Post* After bronchodilator inhalation, *FEV*_*1*_ Forced expiratory volume in 1 s, *FVC* Forced vital capacity, *b* Unstandardized coefficients, *OR* Odds ratios, *CI* Confidence interval

Adjusted model 1: adjusted for age, gender, height, body mass index, education, smoking status, residence, occupational exposure and biomass fuel exposure

Adjusted model 2: additional adjusted for chronic cough during childhood, parental history of respiratory diseases, history of chronic bronchitis, other respiratory diseases and other systemic diseases

**Fig. 2:**
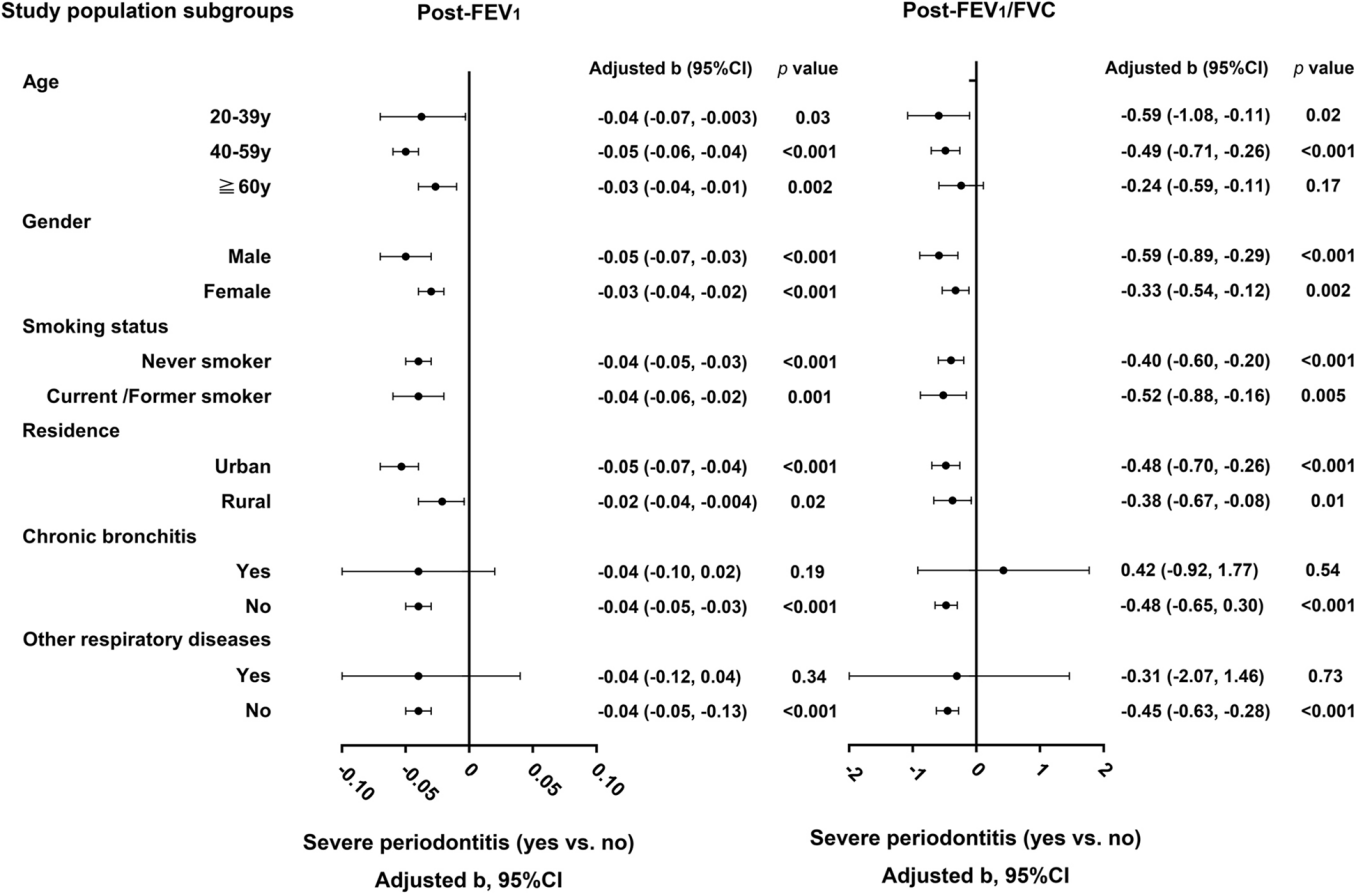
Associations of symptoms of severe periodontitis with post-FEV_1_ and post-FEV_1/_FVC by subgroup multiple linear regression analyses (FEV_1_: forced expiratory volume in 1 s; FVC: forced vital capacity. Other respiratory diseases included chronic obstructive pulmonary disease, emphysema, bronchiectasis, asthma, tuberculosis, idiopathic pulmonary fibrosis and lung cancer. Adjusted model: adjusted for age, gender, height, body mass index, education, smoking status, residence, occupational exposure, biomass fuel exposure, chronic cough during childhood, parental history of respiratory diseases, history of chronic bronchitis, other respiratory diseases and other systemic diseases. When the study population was grouped by a variable, this variable was not included in the adjusted model

For subgroup analyses (Fig. 2), SSP were both negatively associated with post-FEV_1_ and post-FEV_1_/FVC in adjusted multiple linear regression analyses when the participants were stratified by age, gender, education, smoking status and residence separately (all *p* < 0.05) except for post-FEV_1_/FVC in the ≥ 60y group that stratified by age (*p* > 0.05), which is consistent with the results in total participants. When the participants were stratified by history of chronic bronchitis or other respiratory diseases, SSP were only negatively associated with post-FEV_1_ and post-FEV_1_/FVC in participants without respiratory diseases (all *p* < 0.001).

## Discussion

In this large nationally representative sample based cross-sectional study with 49,202 participants, we found that SSP were negatively associated with post-bronchodilator lung function variables FEV_1_, FVC and FEV_1_/FVC and significantly associated with post-FEV_1_/FVC < 0.7 after adjusting for several confounding factors. To the best of our knowledge, this is the largest nationwide study of the general population and the first Chinese population-based study to comprehensively examine the associations between SSP and post-bronchodilator lung function.

The association between periodontitis and respiratory diseases has received much attention in recent decades. Several potential mechanisms have been proposed to explain how a localized inflammatory disease around the teeth can harm lung health, such as attributed to the migration of periodontal pathogens to the lung and hematopoietic transmission of proinflammatory mediators produced in diseased periodontal tissue to the lung [4]. Periodontal pathogens are key factors in the development of periodontitis and accumulate in periodontal environments. As periodontal pathogens may migrate to the airway directly by aspiration or through circulation by entering the capillary from the wound gingival epithelium [16]. These pathogens may cause airway and alveoli infections and inflammation [16, 17]. Inflammation is the key pathological feature of COPD, which may result in remodeling of the airways and destruction of the alveoli, reducing their capacity to expand, and may also increase the mucous secretions that obstruct the airways, thus causing obstructive lung function impairment[5]. Therefore, the migration of periodontal pathogens into the airways and alveoli may contribute to the association between periodontitis and reduced lung function. An intervention study showed that periodontal therapy in COPD patients with periodontitis could improve their lung function [18]. 

FEV_1_ and FEV_1_/FVC are the most commonly used lung function parameters to assess ventilation dysfunction. A longitudinal study in the general Japanese population found that periodontitis was a risk factor for rapid FEV_1_ decline [8]. Two population-based studies showed that reduced FEV_1_/FVC was associated with poor periodontal health [7, 11]. In this study, we found that SSP were negatively associated with post-FEV_1_ and FEV_1_/FVC after adjusted for several confounding factors, which were further confirmed by subgroup analyses. However, when participants were stratified by respiratory diseases, SSP were only significantly associated with post-FEV_1_ and post-FEV_1_/FVC in participants without respiratory diseases. This is likely because severe periodontitis has a weaker effect on lung function than these respiratory diseases do on lung function impairment.

In this study, we found that SSP were significantly associated with post-FEV_1_/FVC < 0.7 in the adjusted models. These results were not consistent with the two previous large population-based studies that more likely used pre-FEV_1_/FVC data [11, 12]. Meanwhile, the different study population and definition of periodontitis may also contribute to the inconsistence of the results. As the post-FEV_1_/FVC < 0.7 is a requirement for COPD diagnosis [13], the results provide evidence for the association between periodontitis and COPD or irreversible airflow obstruction.

Our study has some strengths. First, the participants were enrolled by a multistage stratified cluster sampling procedure from the national population aged 20 to 89 years. The good representation of the study population and the large sample size make the results more reliable. Second, we used post-bronchodilator lung function data to further examine the associations between SSP and lung function.

There are also several limitations in this study. First, this is a cross-sectional study, so a cause and effect relationship can not be established by this study. Second, some participants were excluded due to missing data or unreliable lung function test results, which slightly decreased the good representation of the study population. Third, no clinical data on periodontitis was collected in this study. The CPHS was a nation-wide study on pulmonary health. Clinical examination of periodontal health for the participants was difficult in this study as its large sample size. Therefore, SSP including tooth mobility and natural tooth loss acquired from a questionnaire were used to alternate the periodontal clinical examination. About 22% of the participants had SSP in this study that was a little lower than the prevalence of severe periodontitis of China (about 30%) according to the 4th Chinese national oral health epidemiological survey [2]. In addition, recall bias may affect the validity of SSP. SSP may probably underestimate the real prevalence of severe periodontitis in the participants of the study. Last, some other risk factors for lung function decline, such as air pollution, were not included in this study. This may partially reduce the strength of the conclusions.

In conclusion, this large and nation-wide population based cross-sectional study showed that SSP were negatively associated with post-bronchodilator lung function in the Chinese population. Large-scale and long-term longitudinal cohort studies are needed to confirm these associations in the future.

## Data Availability

The datasets used during the current study are available from the corresponding author.

## Abbreviations

COPD: Chronic obstructive pulmonary disease
FEV_1_: Forced expiratory volume in 1 s
FVC: Forced vital capacity
SSP: Symptoms of severe periodontitis
OR: Odds ratios
CI: Confidence interval
CPHS: China Pulmonary Health Study
GOLD: Global Initiative for Chronic Obstructive Lung Disease
BMI: Body mass index
